# Long Non-coding RNA EPIC1 Promotes Cell Proliferation and Motility and Drug Resistance in Glioma

**DOI:** 10.1016/j.omto.2020.03.011

**Published:** 2020-03-30

**Authors:** Jianjiao Wang, Shuguang Yang, Qiongyu Ji, Qingsong Li, Fenggang Zhou, Yang Li, Fei Yuan, Jie Liu, Yu Tian, Yan Zhao, Yongri Zheng

**Affiliations:** 1 Department of Neurosurgery, the 2nd Affiliated Hospital, Harbin Medical University, Harbin 150086, China; 2 Huxi Hospital Affiliated to Jining Medical College, Shanxian Central Hospital, Heze 274300, China

**Keywords:** glioma, EPIC1, proliferation, Cdc20, invasion, migration, oncogene, non-coding RNA, treatment, cancer

## Abstract

Evidence has revealed that long non-coding RNAs (lncRNAs) are involved in carcinogenesis and tumor progression. lncRNAs play an important role in regulation of numerous cellular processes including cell proliferation, apoptosis, cell cycle, differentiation, and motility. Several studies have demonstrated that lncRNA EPIC1 governs cell growth, cell cycle, migration, invasion, and drug resistance in human malignancies. However, the role of EPIC1 and its underlying molecular mechanisms in glioma have not been investigated. In this study, we determined the function of EPIC1 in glioma cells via upregulation or downregulation of EPIC1. We further dissected the mechanism of EPIC1-mediated tumor progression in glioma. Our results showed that inhibition of EPIC1 suppressed cell viability, induced apoptosis, inhibited cell invasion, and increased cell sensitivity to temozolomide in glioma cells. Consistently, overexpression of EPIC1 exhibited the opposite effects in glioma cells. Moreover, our data suggest that EPIC1 exerts its biological functions via targeting Cdc20 in glioma cells. In line with this, overexpression of Cdc20 reversed the EPIC1-mediated tumor progression in glioma cells. Therefore, targeting EPIC1 might be a useful approach for glioma treatment.

## Graphical Abstract

**Figure:**
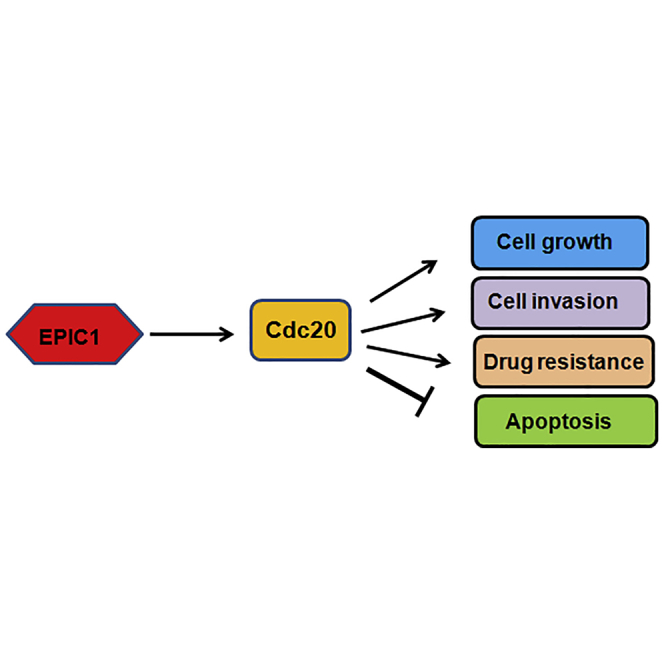

## Teaser

The role of lncRNA EPIC1 in glioma is unclear. This study identified the molecular mechanism of lncRNA EPIC1-mediated tumor progression in glioma. EPIC1 exhibits its functions through targeting Cdc20 in glioma cells. Targeting EPIC1 could be a strategy for treating glioma.

## Introduction

Glioma is the common cancer type in the central nervous system, which has aggressive and high angiogenic feature.^1^ Glioma is one of the common reasons of cancer-related death due to high-grade growth and invasion of glioma cells.^1^ Multiple treatments have been used for the treatment of patients with glioma, such as surgery, radiotherapy, chemotherapy, and combination management.^2^ Glioma is an aggressive malignant tumor, and patients often have a poor prognosis and 5-year survival rate is about 10%.^3^ Temozolomide (TMZ) is one common chemotherapeutic drug for treating glioma in the clinic.^4^^,^^5^ However, glioma patients often obtain the resistance to TMZ during the treatment process.^6^, ^7^, ^8^ Thus, it is essential to discover the compound for glioma therapy to obtain better outcomes via determining the mechanism of glioma genesis and progression.

Long non-coding RNAs (lncRNAs), as part of the non-coding RNA family, have more than 200 nucleotides length.^9^ Due to being without uninterrupted open reading frames, lncRNAs cannot be translated into proteins.^10^ However, lncRNAs could regulate the expression of its downstream proteins, leading to regulation of cellular functions such as cell proliferation, apoptosis, invasion, and metastasis.^11^ Accumulated evidence has unveiled that multiple lncRNAs are involved in glioma genesis and progression.^12^ lncRNAs play an oncogenic or tumor-suppressive role in glioma initiation and progression.^13^ Aberrant expression signatures of lncRNAs have been revealed to be correlated with glioma development and malignant progression.^13^ For example, linc00645 enhanced transforming growth factor beta (TGF-β)-triggered epithelial mesenchymal transition (EMT) through regulation of microRNA-205-3p (miR-205-3p) and zinc finger E-box binding homeobox 1 (ZEB1) in glioma.^14^ Targeting lncRNA MALAT1 (metastasis-associated lung adenocarcinoma transcript-1)/miR-199a/ZHX1 (zinc fingers and homeoboxes) exhibited anti-tumor activities in glioblastoma.^15^ lncRNAs are also key regulators in EMT in glioma, implying that lncRNAs could be involved in cell invasiveness and metastasis in glioma.^16^ lncRNA EPIC1 has been reported to play a critical role in a wide range of human cancers.^17^^,^^18^ However, the function and mechanism of EPIC1 in glioma have not been explored.

In the present study, we aimed to determine the role of EPIC1 in glioma progression. We measured the cell viability by MTT (3-4,5-dimethyl-2- thiazolyl-2, 5-diphenyl-2-H-tetrazolium bromide) in glioma cells after EPIC1 downregulation or overexpression. We further detected the cell apoptosis by ELISA in glioma cells after EPIC1 modulation. Moreover, cell invasive activity was examined by Transwell invasion assay in cells with EPIC1 modulation. In addition, we explored whether EPIC1 is involved in TMZ resistance of glioma cells. Lastly, we intended to dissect the mechanism of EPIC1 in glioma progression. Our study will provide the evidence for the role of EPIC1 in cell viability, apoptosis, invasion, and drug resistance in glioma.

## Results

### Downregulation of lncRNA EPIC1 Suppresses Cell Viability

**Figure 1:**
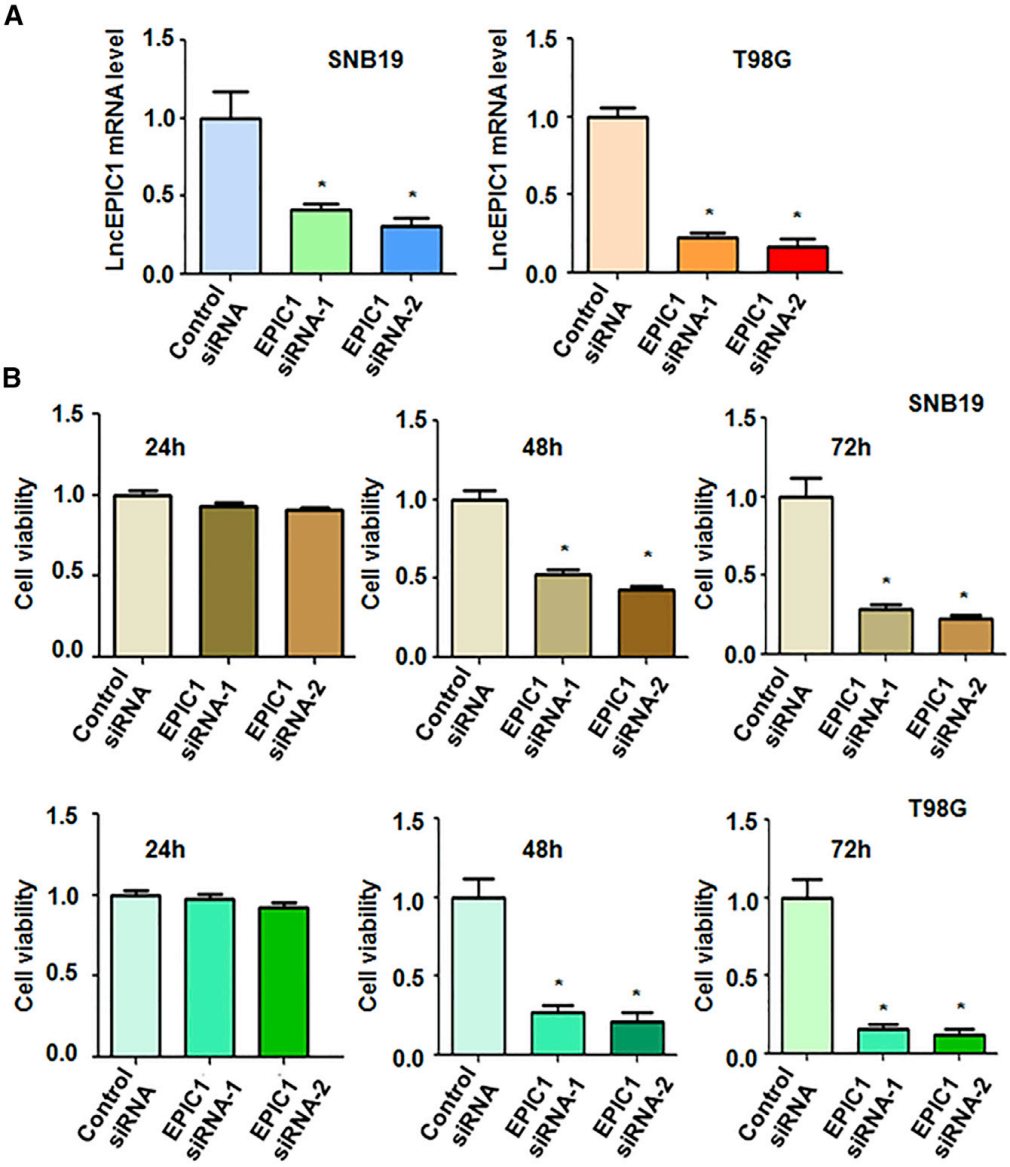
Effect of EPIC1 Downregulation on Cell Viability (A) Real-time PCR was utilized to test EPIC1 expression in glioma cells after EPIC1 siRNA transfection. ∗p < 0.05 versus control siRNA. (B) MTT assay was utilized to measure cell viability in glioma cells after EPIC1 siRNA transfection for 24 h, 48 h, and 72 h.

To determine the role of EPIC1 in glioma cells, we transfected SNB19, T98G, and U97MG cells with EPIC1 small interfering RNA (siRNA). The efficacy of downregulation of EPIC1 by siRNA transfection was measured by reverse transcriptase PCR (RT-PCR). The results from RT-PCR demonstrated that EPIC1 expression level was significantly decreased in three glioma cell lines after EPIC1 siRNA transfection (Figures 1A and S1A). To explore whether EPIC1 controls cell viability in glioma cells, we utilized MTT assay to measure viability of glioma cells after EPIC1 siRNA transfection. Our data from MTT assay showed that downregulation of EPIC1 suppressed cell viability in three glioma cell lines (Figures 1B and S1B). This finding suggests that EPIC1 could play an oncogenic role in cell viability of glioma cells.

### Downregulation of EPIC1 Induces Apoptosis

**Figure 2:**
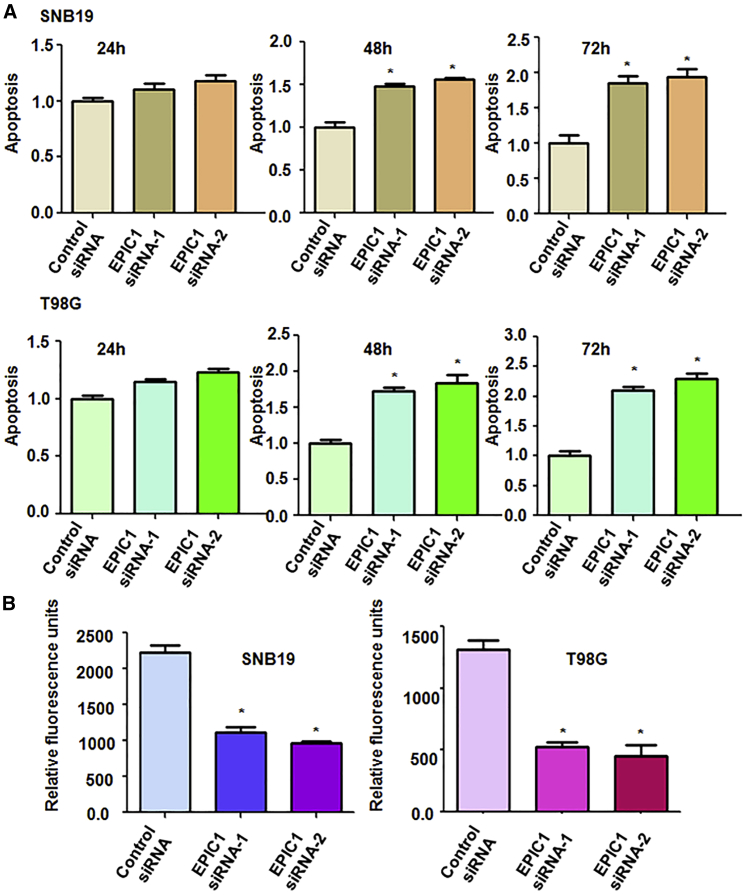
Effect of EPIC1 Downregulation on Apoptosis and Invasion (A) Apoptosis was examined in glioma cells after EPIC1 siRNA transfection. ∗p < 0.05 versus control siRNA. (B) Cell invasion assay was used to test the invasiveness of glioma cells after EPIC1 siRNA transfection.

We explored whether EPIC1 modulation could regulate cell apoptosis in glioma cells. After the SNB19, T98G, and U97MG cells were transfected with EPIC1 siRNAs, the histone DNA apoptosis ELISA assay was utilized to measure the cell apoptotic death in glioma cells. We found that downregulation of EPIC1 induced cell apoptosis in three glioma cell lines (Figures 2A and S1C). This result indicates that downregulation of EPIC1-mediated cell viability inhibition is partly due to induction of cell apoptosis in glioma cells.

### Downregulation of EPIC1 Inhibits Cell Invasiveness

Next, cell invasion is a key cellular process for promotion of tumor metastasis. Thus, we explored whether EPIC1 could be involved in regulation of cell invasion in glioma cells. To achieve this goal, we performed cell invasion assay in three glioma cells after EPIC1 siRNA transfection by Transwell inserts with Matrigel. Our data revealed that EPIC1 downregulation by siRNA transfection suppressed cell invasive activity in three glioma cell lines (Figures 2B and S1D). This result demonstrates that EPIC1 governs cell invasiveness in glioma cells.

### lncRNA EPIC1 Increases Cell Viability

**Figure 3:**
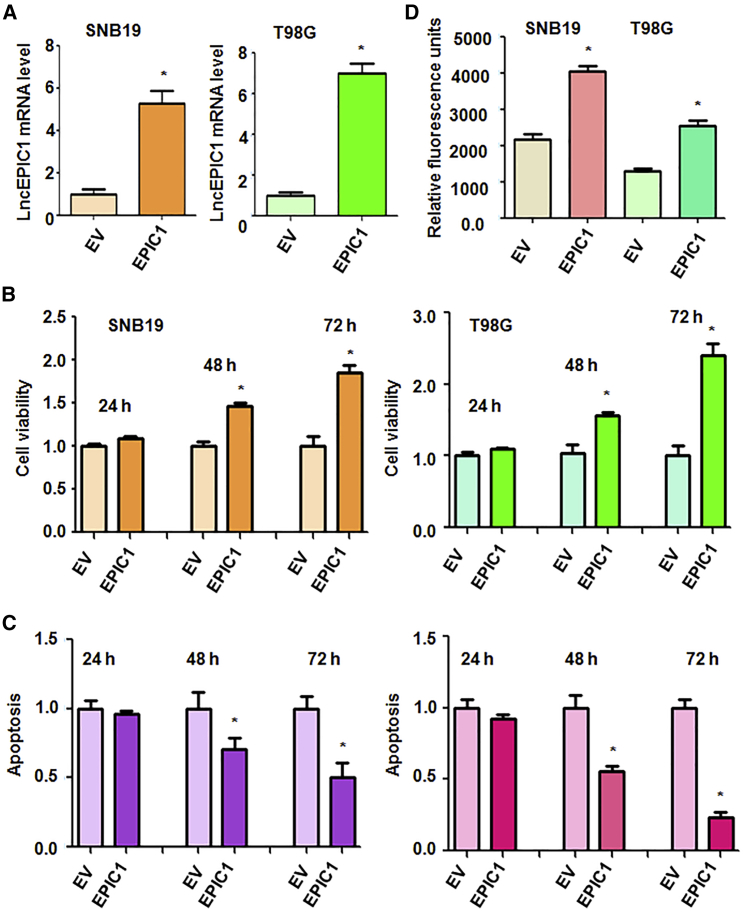
Effect of EPIC1 Overexpression on Cell Viability, Apoptosis, and Invasion (A) Real-time PCR was used to test EPIC1 level in glioma cells after infection with recombinant lentiviruses containing the full length of EPIC1. EV, empty vector. ∗p < 0.05 versus EV. (B) MTT assay was used to test cell viability in glioma cells after EPIC1 overexpression. (C) Apoptosis was tested in glioma cells after overexpression of EPIC1. (D) Cell invasion assay was used to test the invasiveness of glioma cells with EPIC1 overexpression.

To further investigate the role of EPIC1 in glioma, we used recombinant lentiviruses containing the full length of EPIC1 to upregulation of EPIC1 in SNB19, T98G, and U97MG cells. RT-PCR was used to detect the efficacy of EPIC1 infection in glioma cells. Our data showed that EPIC1 expression was remarkably increased after EPIC1 lentivirus infection (Figures 3A and S2A). MTT assay was applied for measurement of cell viability in glioma cells after EPIC1 lentivirus infection. We noticed that overexpression of EPIC1 increased cell viability in three glioma cell lines (Figures 3B and S2B). These data further validate that EPIC1 plays an oncogenic function in cell viability in glioma.

### lncRNA EPIC1 Inhibits Apoptosis and Increases Invasion

To further validate the function of EPIC1 in glioma cells, we measured the cell apoptosis in SNB19, T98G, and U97MG cells after EPIC1 lentivirus infection. Histone DNA apoptosis ELISA was conducted to detect the cell apoptosis in glioma cells with EPIC1 upregulation. We found that overexpression of EPIC1 inhibited cell apoptosis in three glioma cell lines (Figures 3C and S2C). Moreover, cell invasion was also investigated in glioma cells after EPIC1 overexpression. We observed that upregulation of EPIC1 increased cell invasive activity in three glioma cell lines (Figures 3D and S2D). These results clearly suggest that EPIC1 is an oncogenic lncRNA in glioma cells.

### lncRNA EPIC1 Increases TMZ Resistance

**Figure 4:**
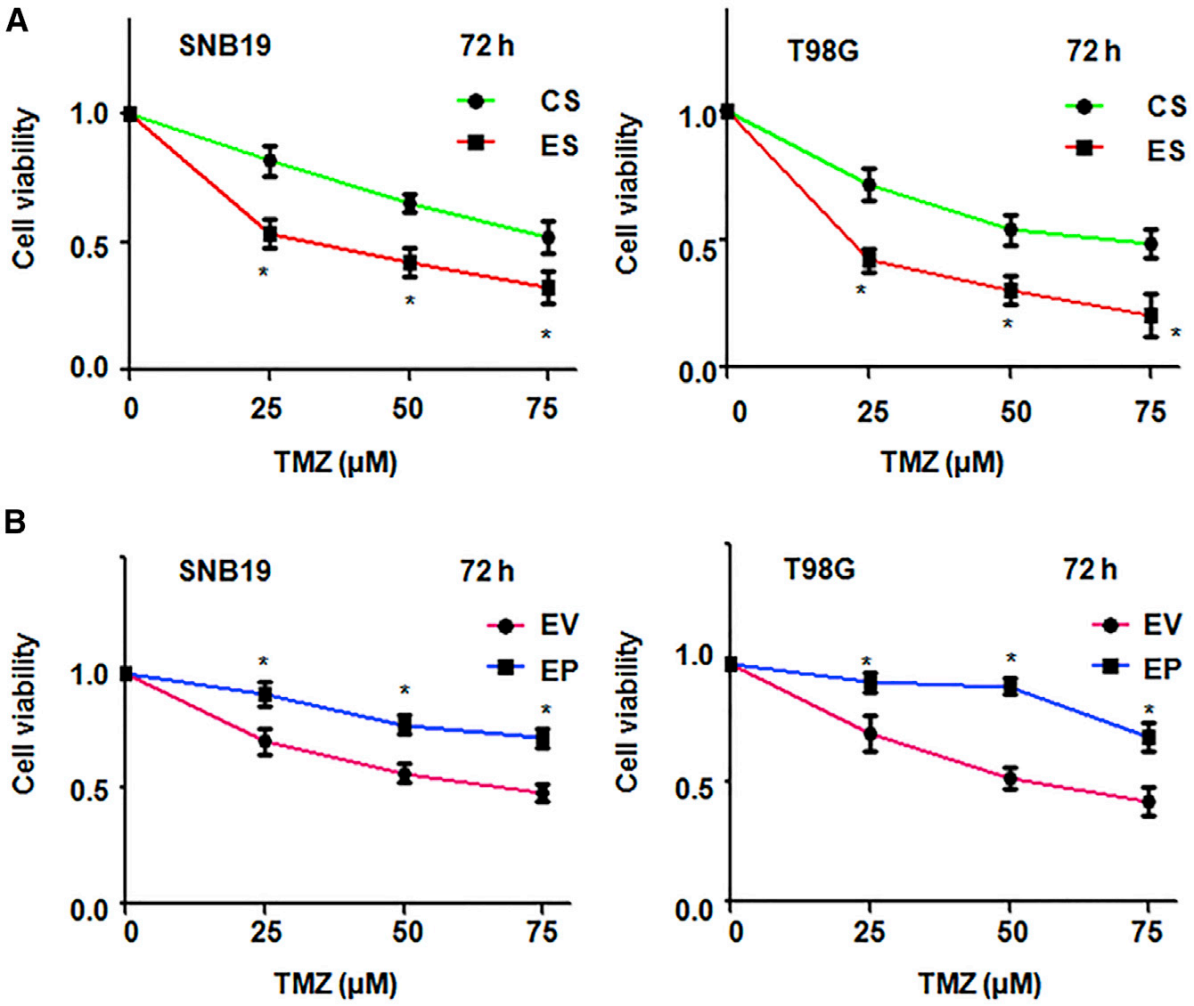
EPIC1 Inhibition Enhances TMZ Sensitivity (A) MTT assay was performed in glioma cells following treatments with EPIC1 siRNA and TMZ exposures. ∗p < 0.05 versus control. (B) MTT assay was utilized in glioma cells after EPIC1 lentivirus infection and TMZ treatment.

To define whether EPIC1 is involved in TMZ resistance in glioma cells, we performed the MTT assay in EPIC1 siRNA or EPIC1 plasmid transfected glioma cells followed by TMZ treatment. We found that EPIC1 siRNA transfection increased glioma cells sensitivity to TMZ treatment (Figures 4A and S3A). In line with this, overexpression of EPIC1 in glioma cells led to resistance to TMA treatment (Figures 4B and S3B). These results suggest that EPIC1 regulates the TMZ resistance in glioma cells, indicating that downregulation of EPIC1 could be a potential approach to overcome TMZ resistance in glioma.

### EPIC1 Regulates Cdc20 Expression

**Figure 5:**
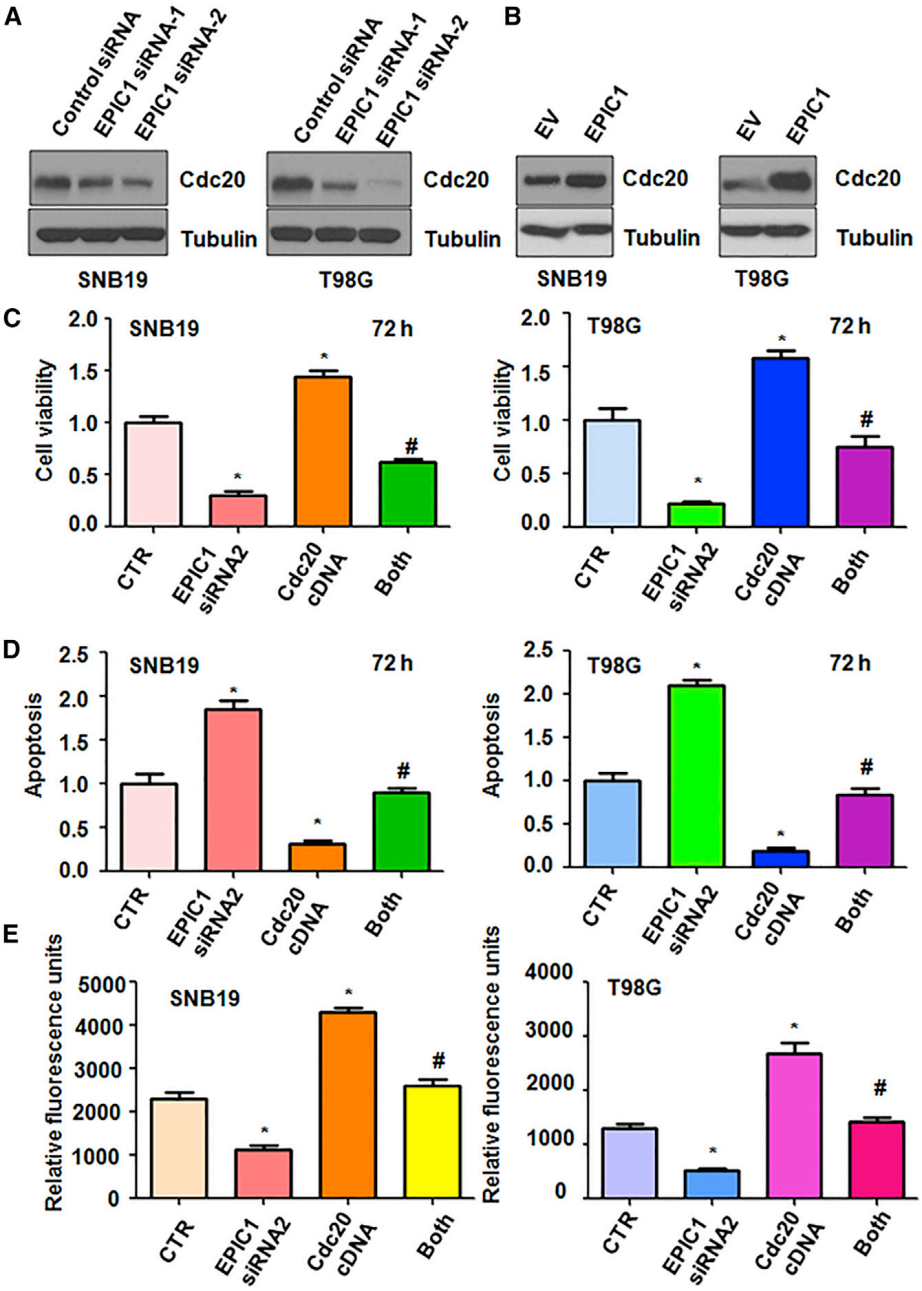
Overexpression of Cdc20 Reverses the EPIC1 siRNA-Mediated Tumor Suppression (A and B) Western blotting analysis was used to measure the expression of Cdc20 in glioma cells after EPIC1 modulation. Inhibition of EPIC1 decreased Cdc20 expression (A), while overexpression of EPIC1 increased Cdc20 level (B). (C) MTT was used to measure viability in glioma cells after EPIC1 siRNA transfection and Cdc20 cDNA transfection. CTR, control. Both: EPIC1 siRNA transfection and Cdc20 cDNA transfection. ∗p < 0.05 versus CTR. #p < 0.05 versus EPIC1 siRNA transfection alone or Cdc20 cDNA transfection alone. (D) Cell apoptosis was performed in glioma cells after EPIC1 inhibition and Cdc20 overexpression. (E) Cell invasion was conducted in glioma cells after EPIC1 suppression and Cdc20 upregulation.

To explore the mechanism of EPIC1-mediated tumorigenesis in glioma cells, we examined the expression level of Cdc20 in SNB19, T98G, and U97MG cells after EPIC1 modulation using western blotting analysis. Our data showed that downregulation of EPIC1 by siRNA transfection reduced the expression of Cdc20 in three glioma cell lines (Figures 5A and S4A). Consistently, overexpression of EPIC1 increased the Cdc20 expression in three glioma cell lines (Figures 5B and S4B).

### Overexpression of Cdc20 Reversed the EPIC1 siRNA-Mediated Tumor Suppression

Next, to further investigate the role of Cdc20 in EPIC1-mediated tumor progression, we overexpressed Cdc20 in glioma cells after EPIC1 siRNA transfection. We found that overexpression of Cdc20 increased cell viability in three glioma cell lines (Figures 5C and S5A). Overexpression of Cdc20 reversed the EPIC1 siRNA-mediated inhibition of cell viability in glioma cells (Figures 5C and S5A). Moreover, Cdc20 overexpression inhibited apoptosis of glioma cells (Figures 5D and S5B). Upregulation of Cdc20 abrogated EPIC1 siRNA-induced apoptosis in three glioma cell lines (Figures 5D and S5B). Furthermore, overexpression of Cdc20 enhanced cell invasion in glioma cells (Figures 5E and S5C). Cdc20 upregulation abolished EPIC1 siRNA-mediated inhibition of cell invasiveness (Figures 5E and S5C). These data indicate that Cdc20 plays an important role in EPIC1-mediated tumor progression.

### Downregulation of Cdc20 Abolishes EPIC1-Mediated Tumor Progression

**Figure 6:**
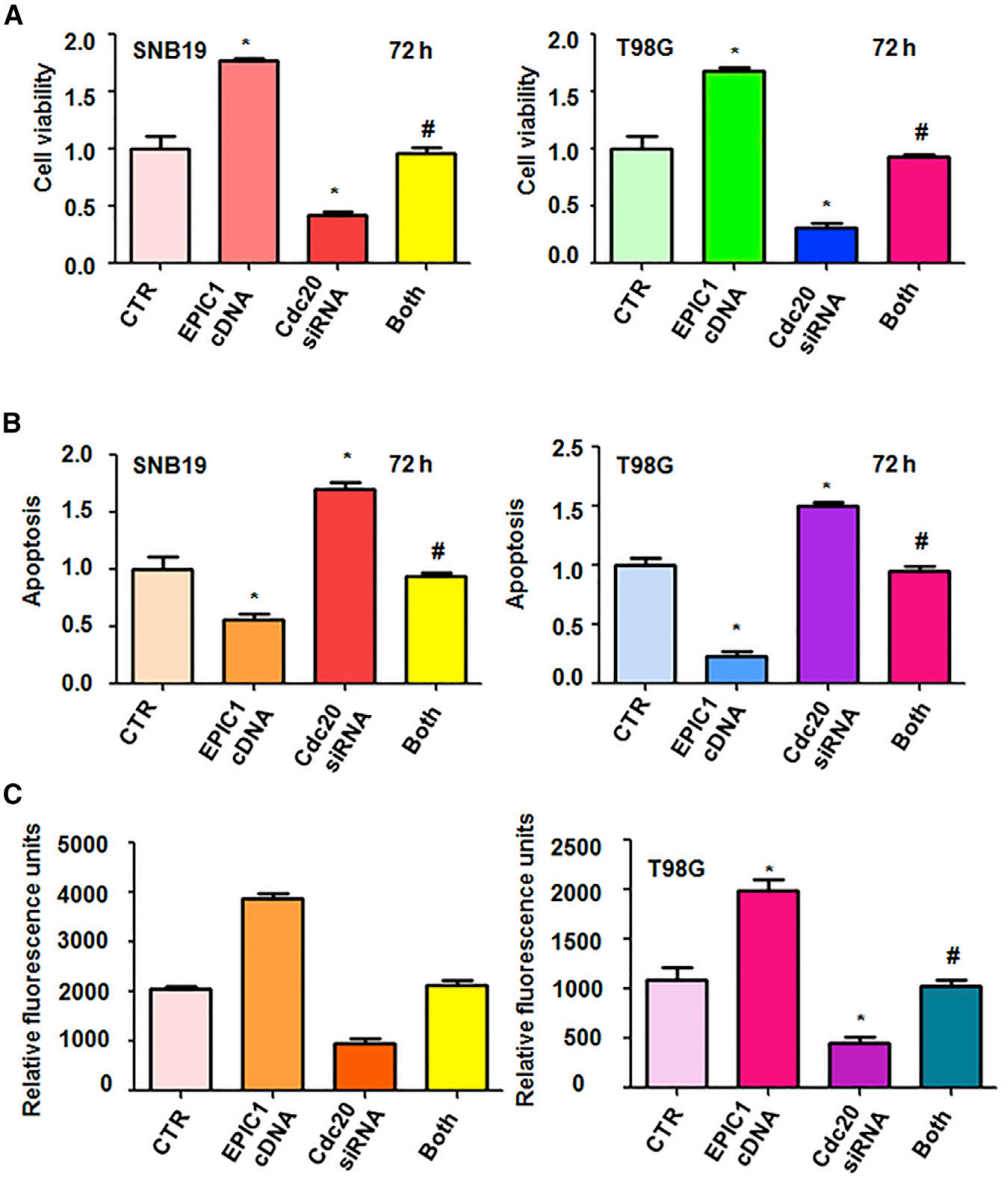
Downregulation of Cdc20 Abolishes EPIC1-Mediated Tumor Progression (A) Cell viability was measured in glioma cells after Cdc20 downregulation and EPIC1 overexpression. CTR, control. Both: EPIC1 cDNA transfection and Cdc20 siRNA transfection. ∗p < 0.05 versus CTR. #p < 0.05 versus EPIC1 cDNA transfection alone or Cdc20 siRNA transfection alone. (B) Cell apoptosis was detested in glioma cells after Cdc20 siRNA transfection and EPIC1 overexpression. (C) Cell invasion was performed in glioma cells after Cdc20 downregulation and EPIC1 upregulation.

The glioma cells were infected with lentiviruses containing the full length of EPIC1 and transfected with Cdc20 siRNA. Then, MTT and apoptosis assays were performed to measure the cell viability and apoptosis in glioma cells. We observed that downregulation of Cdc20 inhibited cell viability in three glioma cell lines (Figures 6A and S5A). Cdc20 downregulation abolished EPIC1-mediated promotion of cell viability (Figures 6A and S5A). Downregulation of Cdc20 induced cell apoptosis in glioma cells and abrogated EPIC1-mediated inhibition of cell apoptosis in glioma (Figures 6B and S5B). Moreover, Cdc20 inhibition retarded cell invasion and abolished EPIC1-mediated enhancement of cell invasive activity in glioma cells (Figures 6C and S5C). These findings indicated that EPIC1 might enhance tumor progression via promotion of Cdc20 in glioma cells.

## Discussion

lncRNAs have been reported to participate in glioma carcinogenesis and progression.^19^ lncRNA RHPN1-AS increased cell proliferation and invasiveness via regulation of miR-625/REG3A in glioma cells.^19^ lncRNA 01494 enhanced cell proliferation, migration, and invasion via regulation of miR-122-5p/CCNG1 axis in glioma.^20^ EPIC1 has been characterized as an oncogenic lncRNA in various types of human cancers. For example, higher expression of EPIC1 is correlated with poor prognosis in breast cancer patients.^17^ Similarly, EPIC1 has been reported to be elevated in human lung cancer tissues and gliomas.^17^^,^^21^ In addition, EPIC1 expression level is elevated in cholangiocarcinoma tissues and several cholangiocarcinoma cancer cell lines when compared with adjacent normal samples and normal immortalized cholangiocyte cells, respectively.^18^ Overexpression of EPIC1 leads to promotion of tumor growth in cell lines and mice.^17^ Downregulation of EPIC1 suppresses cell growth, survival, and proliferation and induces apoptosis and cell-cycle arrest in lung cancer cells.^21^ In line with these reports, EPIC1 controls cell growth, colony formation, cell apoptotic death, and cell-cycle progression in cholangiocarcinoma.^18^ Interestingly, one study found that EPIC1 is downregulated in osteosarcoma cell lines and tissues.^22^ EPIC1 overexpression causes the suppression of cell viability and invasion in osteosarcoma.^22^ EPIC1 also inhibits tumor growth in the osteosarcoma xenograft mouse. The function of EPIC1 in osteosarcoma is conducted via promotion of MEF2D ubiquitylation.^22^ However, the function and mechanism of EPIC1-involved tumorigenesis in glioma is ambiguous. In the present study, we found that overexpression of EPIC1 enhanced the glioma progression, while downregulation of EPIC1 exhibited antitumor activity in glioma, indicating that EPIC1 plays an oncogenic role in glioma.

EPIC1 enhances cell-cycle progression via interaction with Myc.^17^ EPIC1 downregulation decrease the expression of Cyclin A1, Cdc20, and Cdc45 in lung cancer cells.^21^ Overexpression of EPIC1 increased these genes’ expressions, leading to cell growth promotion in lung cancer cells.^21^ Furthermore, downregulation of EPIC1 leads to inhibition of Cyclin A and Cyclin D and CDK9 in cholangiocarcinoma cells.^18^ Here, we found that EPIC1 downregulation inhibited the expression of Cdc20, while overexpression of EPIC1 elevated Cdc20 expression level in glioma. Cdc20 has been reported to be widely expressed in glioblastomas tissues.^23^ One study showed that Cdc20 was preferentially existed in tumorigenic glioma tumor initiating cells (TICs). Inhibition of Cdc20 repressed proliferation, self-renewal of TICs, and tumor growth in mice via apoptosis induction and suppression of cell-cycle progression and disruption of p21.^24^ Similarly, Cdc20 positively governed invasion and self-renewal in glioblastoma stem-like cells (GSCs) via regulation of SOX2.^25^

EPIC1 has been found to promote the cell resistance to iBET762 (bromodomain and extra-terminal motif inhibitor) and JQ-1 via activation of MYC transcriptional activity in cancer cells,^26^ suggesting that EPIC1 could be useful as a predictive lncRNA for BET inhibitors. Our previous study has shown that Cdc20 knockdown in TMZ-resistant cells increased the sensitivity of cells to TMZ in glioma cells.^27^ Overexpression of Cdc20 was observed in TMZ-resistant cells, which obtained EMT features.^27^ Our results also showed that EPIC1 overexpression increased the resistance of glioma cells to TMZ. Moreover, depletion of Cdc20 abolished EPIC1-mediated TMZ resistance in glioma cells. Consistently, overexpression of Cdc20 abrogated TMZ sensitivity due to downregulation of EPIC1 in glioma cells. Thus, inhibition of EPIC1 or Cdc20 could be a potential strategy for overcoming the resistance to TMZ in glioma patients.

## Materials and Methods

### Reagents and Antibodies

Dulbecco’s modified Eagle’s medium (DMEM) was bought from GIBCO. TMZ and MTT were obtained from Sigma-Aldrich (St. Louis, MO, USA). TRIzol and lipofectamine 2000 were purchased from Invitrogen (Carlsbad, CA, USA). Primary antibodies for Cdc20 and tubulin were bought from Cell Signaling Technology (Danvers, MA, USA).

### Cell Culture

The SNB19 and T98G human glioma cell lines were bought from the Chinese Academy of Sciences (Shanghai, China). These cells were maintained in DMEM with 10% FBS and 1% penicillin/streptomycin at 37°C with 5% CO_2_. These cells were transfected with lnc EPIC1 siRNAs or lnc EPIC1 plasmid.

### Transfection

The SNB19 and T98G human glioma cell lines were seeded in 6-well tissue culture plates and transfected with lnc EPIC1 siRNAs or the scrambled siRNA by lipofectamine 2000. The recombinant lentiviruses containing full-length EPIC1 and the nonspecific control as empty vector (EV) were synthesized by Genechem Company (Shanghai, China).

### Quantitative Real-Time PCR

Total RNA in glioma cells was extracted using TRIzol reagent. Then, real-time RT-PCR was performed as described before. The primer sequences were described as following: EPIC1: forward 5′-TAT CCC TCA GAG CTC CTG CT-3′, reverse 5′-AGG CTG GCA AGT GTG AAT CT-3′.

### MTT Assays

The transfected SNB19 and T98G cells (2.5 × 10^3^ cells/well) were seeded in 96-well plates for incubation with different times. Then MTT assays were used to measure cell viability as described previously.^27^

### Histone DNA Apoptosis ELISA Assay

The transfected SNB19 and T98G cells were seeded in 6-well plates for different times. The histone DNA apoptosis ELISA kit (Roche, Shanghai, China) was conducted to measure cell apoptosis according to the manufacturer’s instructions. The optical density (OD) values at 405 nm were determined.

### Transwell Invasion Assay

The transfected SNB19 and T98G cells were seeded in the upper chamber of the Matrigel precoated Transwell inserts for 20 h. To quantify the invasion of glioma cells through the basement membrane of Transwell matrigel, we performed the CytoSelect 96-well cell invasion assay (Cell Biolabs, San Diego, CA, USA) according to the manufacturer’s protocol.

### Western Blotting Analysis

The transfected SNB19 and T98G cells were seeded in 10 cm dishes for different times. The cells were lysed using cell lysis buffer (Solarbio, Beijing, China). The protein concentrations were determined by bicinchoninic acid (BCA) protein assay. The proteins were boiled and separated on SDS-PAGE and then were transferred onto polyvinylidene fluoride (PVDF) membrane. Western blotting assay was performed as described previously.^28^

### Statistical Analysis

GraphPad Prism 5.0 (Graph Pad Software, La Jolla, CA, USA) was utilized to analyze the data. The Student’s t test and ANOVA were applied for data analysis. Data presented were expressed as mean ± standard deviations (SDs). Values of p < 0.05 were considered as a significant difference.

## Author Contributions

J.W. designed and performed the experiments, analyzed the data, wrote the manuscript. S.Y., Q.J., Q.L., and F.Z., performed the experiments. Y.L., F.Y., J.L., Y.T., Y. Zhao, and Y. Zheng analyzed the data.

## Conflicts of Interest

The authors declare no competing interests.

## Supplemental Information

Document S1. Figures S1–S5

Document S2. Article plus Supplemental Information

## References

[1] Siegel R.L., Miller K.D., Jemal A. (2019). Cancer statistics, 2019. CA Cancer J. Clin..

[2] Alinezhad A., Jafari F. (2019). Novel management of glioma by molecular therapies, a review article. Eur. J. Transl. Myol..

[3] Di Carlo D.T., Cagnazzo F., Benedetto N., Morganti R., Perrini P. (2019). Multiple high-grade gliomas: epidemiology, management, and outcome. A systematic review and meta-analysis. Neurosurg. Rev..

[4] Daniel P., Sabri S., Chaddad A., Meehan B., Jean-Claude B., Rak J., Abdulkarim B.S. (2019). Temozolomide Induced Hypermutation in Glioma: Evolutionary Mechanisms and Therapeutic Opportunities. Front. Oncol..

[5] Bahadur S., Sahu A.K., Baghel P., Saha S. (2019). Current promising treatment strategy for glioblastoma multiform: A review. Oncol. Rev..

[6] Jiapaer S., Furuta T., Tanaka S., Kitabayashi T., Nakada M. (2018). Potential Strategies Overcoming the Temozolomide Resistance for Glioblastoma. Neurol. Med. Chir. (Tokyo).

[7] Taylor M.A., Das B.C., Ray S.K. (2018). Targeting autophagy for combating chemoresistance and radioresistance in glioblastoma. Apoptosis.

[8] Grek C.L., Sheng Z., Naus C.C., Sin W.C., Gourdie R.G., Ghatnekar G.G. (2018). Novel approach to temozolomide resistance in malignant glioma: connexin43-directed therapeutics. Curr. Opin. Pharmacol..

[9] Huarte M. (2015). The emerging role of lncRNAs in cancer. Nat. Med..

[10] Wahlestedt C. (2013). Targeting long non-coding RNA to therapeutically upregulate gene expression. Nat. Rev. Drug Discov..

[11] Yao R.W., Wang Y., Chen L.L. (2019). Cellular functions of long noncoding RNAs. Nat. Cell Biol..

[12] Wang L., He Z. (2019). Functional Roles of Long Non-Coding RNAs (LncRNAs) in Glioma Stem Cells. Med. Sci. Monit..

[13] Zhang X.Q., Leung G.K. (2014). Long non-coding RNAs in glioma: functional roles and clinical perspectives. Neurochem. Int..

[14] Li C., Zheng H., Hou W., Bao H., Xiong J., Che W., Gu Y., Sun H., Liang P. (2019). Long non-coding RNA linc00645 promotes TGF-β-induced epithelial-mesenchymal transition by regulating miR-205-3p-ZEB1 axis in glioma. Cell Death Dis..

[15] Liao K., Lin Y., Gao W., Xiao Z., Medina R., Dmitriev P., Cui J., Zhuang Z., Zhao X., Qiu Y. (2019). Blocking lncRNA MALAT1/miR-199a/ZHX1 Axis Inhibits Glioblastoma Proliferation and Progression. Mol. Ther. Nucleic Acids.

[16] Xin S., Huang K., Zhu X.G. (2019). Non-coding RNAs: Regulators of glioma cell epithelial-mesenchymal transformation. Pathol. Res. Pract..

[17] Wang Z., Yang B., Zhang M., Guo W., Wu Z., Wang Y., Jia L., Li S., Xie W., Cancer Genome Atlas Research Network (2018). lncRNA Epigenetic Landscape Analysis Identifies EPIC1 as an Oncogenic lncRNA that Interacts with MYC and Promotes Cell-Cycle Progression in Cancer. Cancer Cell.

[18] Li Y., Cai Q., Li W., Feng F., Yang L. (2018). Long non-coding RNA EPIC1 promotes cholangiocarcinoma cell growth. Biochem. Biophys. Res. Commun..

[19] Cui P., Su J., Li Q., Xu G., Zhu N. (2019). LncRNA RHPN1-AS1 Targeting miR-625/REG3A Promotes Cell Proliferation And Invasion Of Glioma Cells. OncoTargets Ther..

[20] Li C., Hu G., Wei B., Wang L., Liu N. (2019). lncRNA LINC01494 Promotes Proliferation, Migration And Invasion In Glioma Through miR-122-5p/CCNG1 Axis. OncoTargets Ther..

[21] Zhang B., Lu H.Y., Xia Y.H., Jiang A.G., Lv Y.X. (2018). Long non-coding RNA EPIC1 promotes human lung cancer cell growth. Biochem. Biophys. Res. Commun..

[22] Zhao W., Zhang D., Qin P., Zhang J., Cui X., Gao J., Wang J., Li J. (2019). Long non-coding RNA EPIC1 inhibits viability and invasion of osteosarcoma cells by promoting MEF2D ubiquitylation. Int. J. Biol. Macromol..

[23] Marucci G., Morandi L., Magrini E., Farnedi A., Franceschi E., Miglio R., Calò D., Pession A., Foschini M.P., Eusebi V. (2008). Gene expression profiling in glioblastoma and immunohistochemical evaluation of IGFBP-2 and CDC20. Virchows Arch..

[24] Xie Q., Wu Q., Mack S.C., Yang K., Kim L., Hubert C.G., Flavahan W.A., Chu C., Bao S., Rich J.N. (2015). CDC20 maintains tumor initiating cells. Oncotarget.

[25] Mao D.D., Gujar A.D., Mahlokozera T., Chen I., Pan Y., Luo J., Brost T., Thompson E.A., Turski A., Leuthardt E.C. (2015). A CDC20-APC/SOX2 Signaling Axis Regulates Human Glioblastoma Stem-like Cells. Cell Rep..

[26] Wang Y., Wang Z., Xu J., Li J., Li S., Zhang M., Yang D. (2018). Systematic identification of non-coding pharmacogenomic landscape in cancer. Nat. Commun..

[27] Wang J., Zhou F., Li Y., Li Q., Wu Z., Yu L., Yuan F., Liu J., Tian Y., Cao Y. (2017). Cdc20 overexpression is involved in temozolomide-resistant glioma cells with epithelial-mesenchymal transition. Cell Cycle.

[28] Wang J., Yang Y., Cao Y., Tang X. (2019). miR-342 inhibits glioma cell proliferation by targeting GPRC5A. Mol. Med. Rep..

