# Supplementary material for: Long Non-coding RNA EPIC1 Promotes Cell Proliferation and Motility and Drug Resistance in Glioma
Source: Mol Ther Oncolytics. 2020 Mar 30;17:130–7. doi: 10.1016/j.omto.2020.03.011 (PMC7163045; doi:10.1016/j.omto.2020.03.011)
Supplement: Document S1. Figures S1–S5 [file mmc1.pdf]

OMTO, Volume *17*

Supplemental Information

Long Non-coding RNA EPIC1 Promotes Cell Proliferation and Motility and Drug Resistance in Glioma

Jianjiao Wang, Shuguang Yang, Qiongyu Ji, Qingsong Li, Fenggang Zhou, Yang Li, Fei Yuan, Jie Liu, Yu Tian, Yan Zhao, and Yongri Zheng

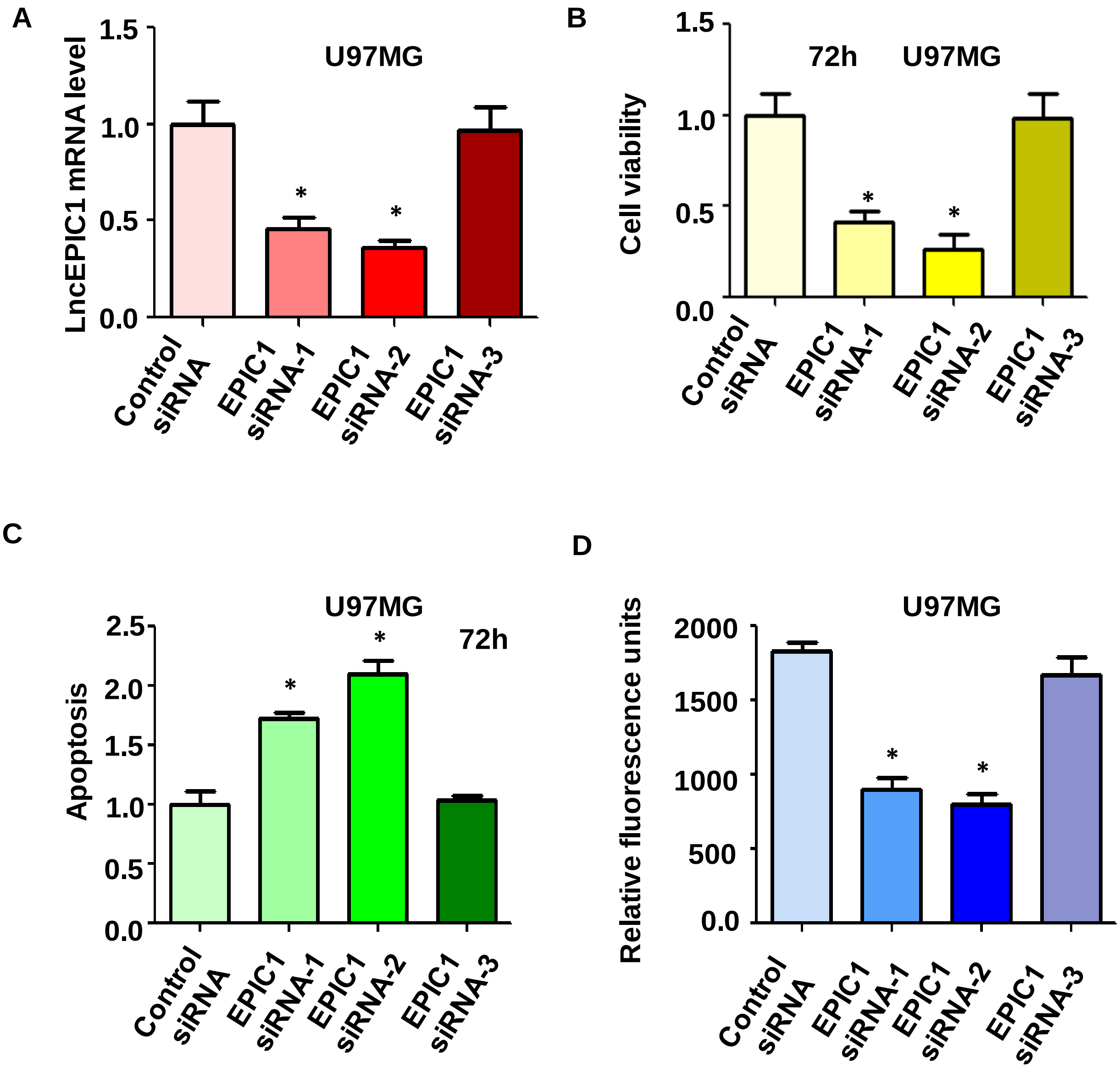

Supplementary Figure 1: Effect of EPIC1 downregulation on cell viability, apoptosis and invasion in U97MG cells.

A, Real-time PCR was utilized to test EPIC1 expression in U97MG cells after EPIC1 siRNA transfection.

B, MTT assay was utilized to measure cell viability in U97MG cells after EPIC1 siRNA transfection for 72h.

C, Apoptosis was examined in U97MG cells after EPIC1 siRNA transfection.

D, Cell invasion assay was used to test the invasiveness of U97MG cells after EPIC1 siRNA transfection . * $p<0.05$ vs control siRNA.

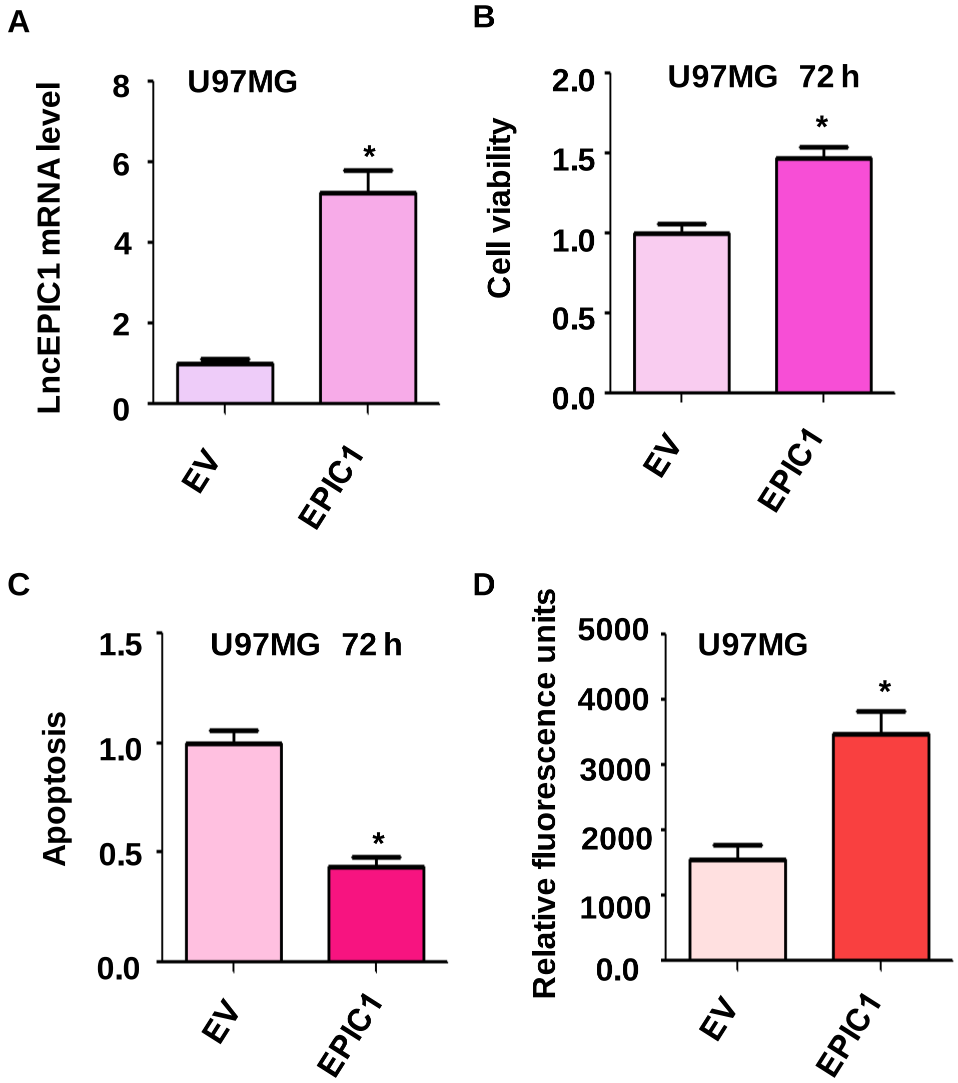

Supplementary Figure 2: Effect of EPIC1 overexpression on cell viability, apoptosis and invasion in U97MG cells.

A, Real-time PCR was used to test EPIC1 level in U97MG cells after infection with recombinant lentiviruses containing full length of EPIC1. EV: empty vector.

B, MTT assay was used to test cell viability in U97MG cells after EPIC1 overexpression.

C, Apoptosis was tested in U97MG cells after overexpression of EPIC1.

D, Cell invasion assay was used to test the invasiveness of U97MG cells with EPIC1 overexpression. * $p<0.05$ vs EV.

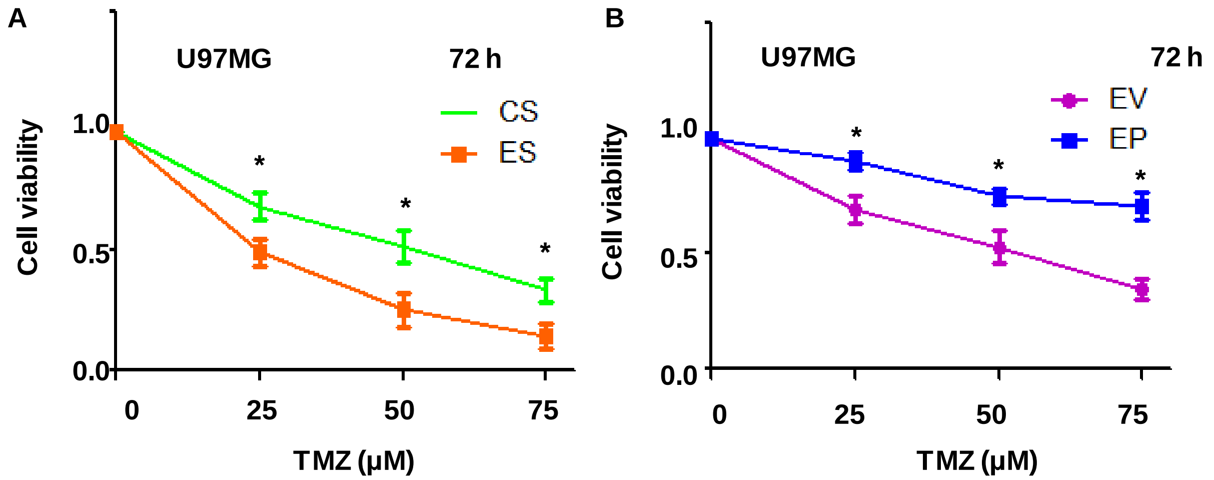

Supplementary Figure 3: EPIC1 inhibition enhances TMZ sensitivity.

A. MTT assay was performed in U97MG cells following treatments with EPIC1 siRNA and TMZ exposures.
B. MTT assay was utilized in U97MG cells after EPIC1 lentivirus infection and TMZ treatment.
* $p<0.05$ vs control.

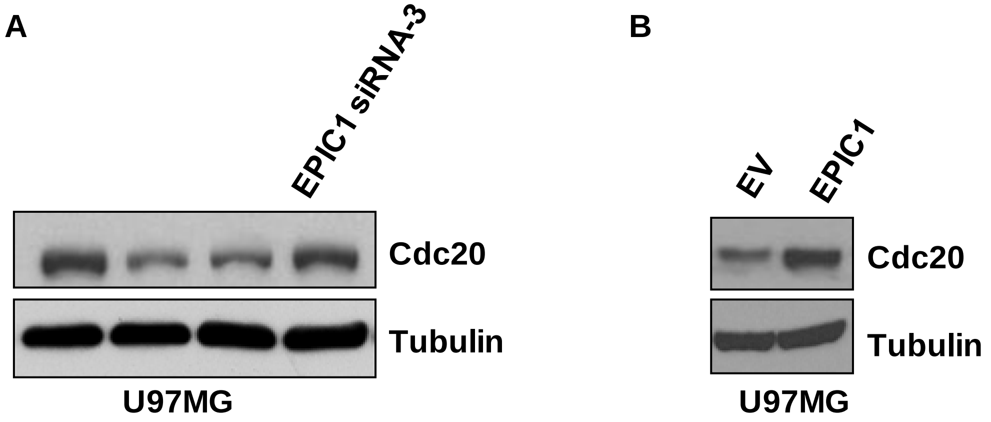

Supplementary Figure 4: LncRNA EPIC1 affects the expression of Cdc20 in U97MG cells.
A, Western blotting analysis was used to measure the expression of Cdc20 in U97MG cells after EPIC1 downregulation.
B, Western blotting analysis was used to measure the expression of Cdc20 in U97MG cells after EPIC1 overexpression.

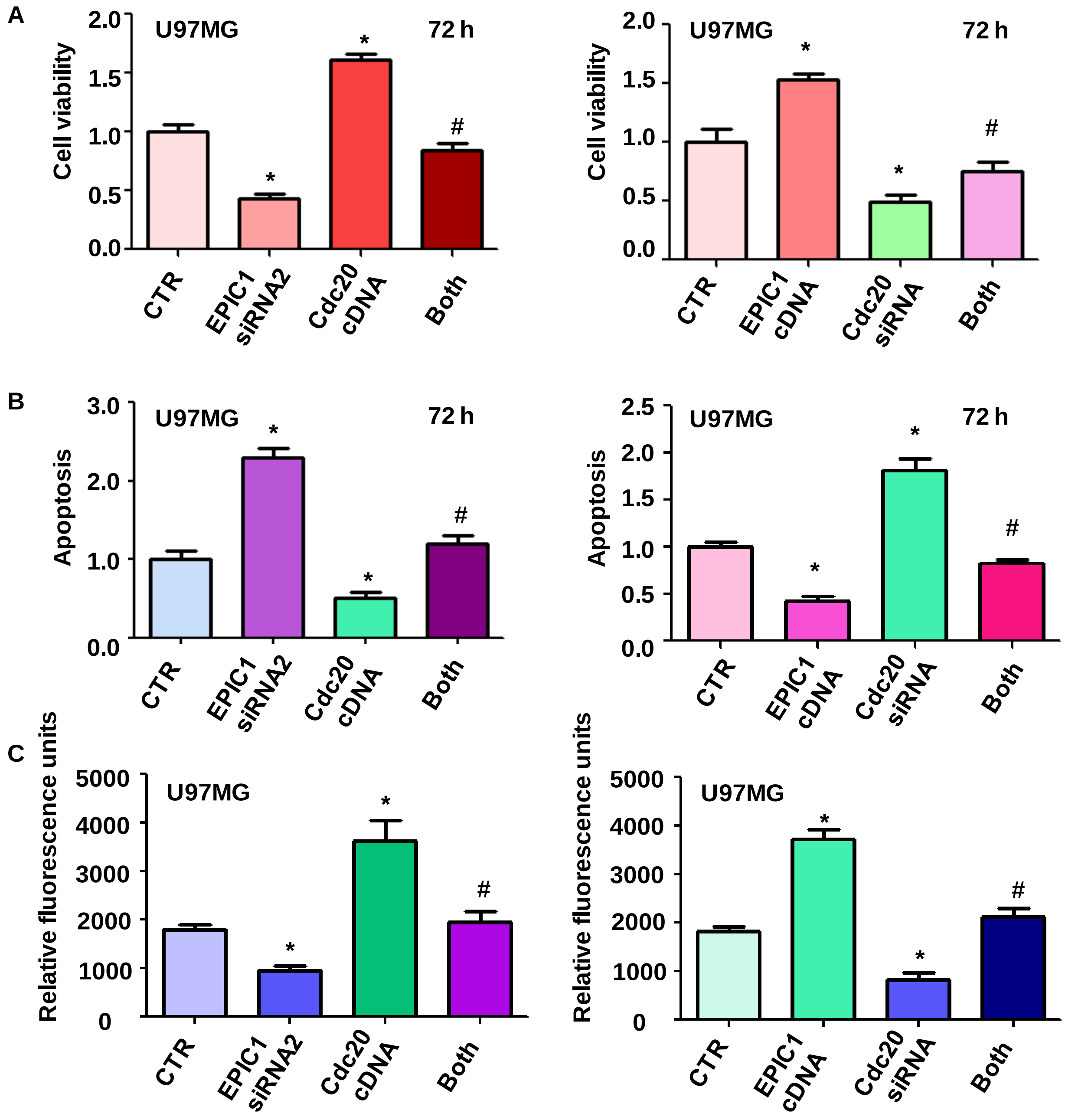

Supplementary Figure 5: Overexpression of Cdc20 reverses the EPIC1 siRNA-mediated tumor suppression.

A, MTT was used to measure viability in U97MG cells after modulation of EPIC1 and Cdc20.
B, Cell apoptosis was performed in U97MG cells after modulation of EPIC1 and Cdc20.
C, Cell invasion was conducted in U97MG cells after modulation of EPIC1 and Cdc20.
CTR: Control. Both: EPIC1 change and Cdc20 change. * p<0.05 vs CTR. #p<0.05 vs EPIC1 change alone or Cdc20 change alone.
